# The Processing of Audiovisual Speech Is Linked with Vocabulary in Autistic and Nonautistic Children: An ERP Study

**DOI:** 10.3390/brainsci13071043

**Published:** 2023-07-08

**Authors:** Kacie Dunham-Carr, Jacob I. Feldman, David M. Simon, Sarah R. Edmunds, Alexander Tu, Wayne Kuang, Julie G. Conrad, Pooja Santapuram, Mark T. Wallace, Tiffany G. Woynaroski

**Affiliations:** 1 Vanderbilt Brain Institute, Vanderbilt University, Nashville, TN 37232, USAmark.wallace@vanderbilt.edu (M.T.W.); tiffany.g.woynaroski@vumc.org (T.G.W.); 2 Department of Hearing and Speech Sciences, Vanderbilt University, Nashville, TN 37232, USA; 3 Frist Center for Autism and Innovation, Vanderbilt University, Nashville, TN 37232, USA; j.i.feldman@vumc.org; 4 Department of Hearing and Speech Sciences, Vanderbilt University Medical Center, Nashville, TN 37232, USA; 5 Department of Psychology, University of Washington, Seattle, WA 98195, USA; 6 Department of Psychology, University of South Carolina, Columbia, SC 29208, USA; 7 Department of Educational Studies, University of South Carolina, Columbia, SC 29208, USA; 8 Neuroscience Undergraduate Program, Vanderbilt University, Nashville, TN 37232, USA; 9 Department of Otolaryngology and Communication Sciences, Medical College of Wisconsin, Milwaukee, WI 53226, USA; 10 Department of Pediatrics, Los Angeles General Medical Center, Keck School of Medicine of University of Southern California, Los Angeles, CA 90033, USA; 11 College of Medicine, University of Illinois Hospital, Chicago, IL 60612, USA; 12 Department of Anesthesiology, Columbia University Irving Medical Center, New York City, NY 10032, USA; 13 Vanderbilt Kennedy Center, Vanderbilt University Medical Center, Nashville, TN 37232, USA; 14 Department of Psychology, Vanderbilt University, Nashville, TN 37232, USA; 15 Department of Pharmacology, Vanderbilt University, Nashville, TN 37232, USA; 16 Department of Psychiatry and Behavioral Sciences, Vanderbilt University Medical Center, Nashville, TN 37232, USA; 17 Department of Communication Sciences and Disorders, John A. Burns School of Medicine, University of Hawaii at Manoa, Honolulu, HI 96813, USA

**Keywords:** speech, sensory, audiovisual, autism, EEG, language, vocabulary

## Abstract

Explaining individual differences in vocabulary in autism is critical, as understanding and using words to communicate are key predictors of long-term outcomes for autistic individuals. Differences in audiovisual speech processing may explain variability in vocabulary in autism. The efficiency of audiovisual speech processing can be indexed via amplitude suppression, wherein the amplitude of the event-related potential (ERP) is reduced at the P2 component in response to audiovisual speech compared to auditory-only speech. This study used electroencephalography (EEG) to measure P2 amplitudes in response to auditory-only and audiovisual speech and norm-referenced, standardized assessments to measure vocabulary in 25 autistic and 25 nonautistic children to determine whether amplitude suppression (a) differs or (b) explains variability in vocabulary in autistic and nonautistic children. A series of regression analyses evaluated associations between amplitude suppression and vocabulary scores. Both groups demonstrated P2 amplitude suppression, on average, in response to audiovisual speech relative to auditory-only speech. Between-group differences in mean amplitude suppression were nonsignificant. Individual differences in amplitude suppression were positively associated with expressive vocabulary through receptive vocabulary, as evidenced by a significant indirect effect observed across groups. The results suggest that efficiency of audiovisual speech processing may explain variance in vocabulary in autism.

## 1. Introduction

Autism is characterized by differences in social communication, by the presence of restricted interests and repetitive patterns of behavior, and by sensory alterations [1]. This neurodevelopmental condition can come at a high personal and economic cost, in large part due to its potential impacts on social, academic, and vocational outcomes of affected individuals [2,3]. The long-term outcomes of autistic individuals are highly heterogenous and have been repeatedly linked with the ability to understand and use words to communicate (i.e., receptive and expressive vocabulary) by school age [2,4,5,6,7]. As a result, there is increasing interest in identifying factors that can explain individual differences in vocabulary in autistic children. Although several behavioral factors that explain variability in vocabulary and broader spoken language ability in autistic children have been identified, e.g., in [8,9], much of the variance remains unexplained. Thus, there is a pressing need to identify additional factors, particularly biobehavioral factors [10], that may improve upon our present ability to explain individual differences in vocabulary in autism.

Individual differences in receptive and expressive vocabulary in autistic children may be explained, at least in part, by individual differences in audiovisual speech processing, an ability that has been theoretically and empirically linked with language development [11,12,13,14,15]. Speech processing is inherently a complex multisensory operation, wherein visual cues from the moving face and mouth complement auditory speech information [16,17,18]. Altered processing of audiovisual speech has been theorized to have downstream and cascading effects on the development of higher-order skills such as receptive and expressive vocabulary and broader language skills [19,20]. Indeed, studies employing behavioral measures (e.g., psychophysics and eye-tracking tasks) have provided preliminary support for associations between audiovisual processing and perception and language and communication in autistic children and in other clinical populations [21,22,23,24,25]. However, no prior studies have employed neural measures of audiovisual speech processing to test the theorized link between audiovisual speech processing and vocabulary in school-age autistic children. Doing so has the potential to provide further insight into the brain basis of phenotypic variance in receptive and expressive vocabulary, and to advance our understanding of the broad range of individual differences in the ability to understand and use words across autistic children.

Visual speech cues, as a source of multisensory redundancy for speech, can influence speech processing in several ways. Nonautistic children appear to use visual speech cues to aid speech perception and language learning beginning in infancy, e.g., in [26,27,28] and continue to integrate auditory and visual speech cues to boost speech perception in challenging listening conditions, such as in noisy environments or when encountering an unfamiliar language, across the lifespan [29,30]. Even into adulthood, having access to and integrating auditory and visual cues leads to improved perceptual accuracy and efficiency of speech perception in a variety of contexts, e.g., in [17,31,32,33,34]. However, prior work indicates that autistic children are highly variable in their perception of visual speech cues and audiovisual speech [14,35,36,37,38,39]. Audiovisual integration in autistic individuals has been repeatedly linked with core and related features of autism, including social communication and language (for a review, see [15]). Thus, it has been proposed that audiovisual speech processing and perception may account for individual differences in language in autistic children, e.g., in [35,36,37,38,40].

### 1.1. Research Background

Speech perception likely relies on audiovisual speech processing efficiency [16]. Audiovisual speech processing efficiency can be measured using electroencephalography (EEG) and has been quantified as the difference in the amplitude of the event-related potential (ERP) in response to audiovisual speech versus auditory-only speech [41]. Specifically, evidence from EEG studies in nonautistic adults shows that, when visual speech cues are present in addition to auditory-only speech cues, ERP amplitudes are suppressed, or reduced, particularly at the P2 component, manifesting as a smaller amplitude for this positive peak that occurs about 200 milliseconds poststimulus and is purported to reflect early perceptual processing for both auditory and visual stimuli [41,42,43]. This phenomenon is known as P2 amplitude suppression and is thought to reflect improved efficiency in stimulus response [41] based on previously observed subadditive effects indicative of the integration of the auditory and visual speech stimuli [44,45,46].

**Figure 1:**
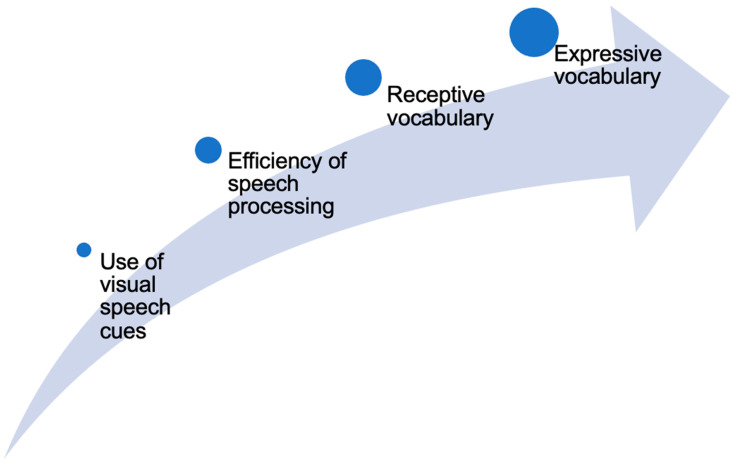
Overview of the theorized cascading effects of audiovisual speech processing on vocabulary. Alterations in sensory processing have been hypothesized to cascade onto higher-order skills, including language. In this example, differences in looking to and processing audiovisual speech may lead to, or explain variability in, the understanding and use of words (i.e., in receptive and expressive vocabulary).

P2 amplitude suppression has been previously observed in both nonautistic children and adults [41,44,47,48,49]. At present, however, it is unclear whether autistic children also display amplitude suppression for audiovisual speech, or if amplitude suppression can explain variability in their vocabulary. As behavioral tasks suggest that autistic children, on average, tend to access visual speech cues less frequently, identify visual speech cues less accurately, and integrate auditory and visual speech cues less effectively than nonautistic peers [36,50,51], we hypothesize that they may also process audiovisual speech less efficiently at the neural level. Such reductions in speech processing efficiency may impact autistic children’s ability to parse the incoming speech stream and to extract linguistic information accurately and efficiently, thereby leading to difficulties with the understanding of words. Sustained difficulties with understanding of words, over the long term, may translate to difficulties with the use or production of words (see Figure 1).

A potential approach for evaluating the theorized cascading effects of speech processing onto the understanding and use of words is mediation analysis, which evaluates direct and indirect effects of a putative predictor on a dependent variable [52]. Based on the hypothesis that reduced speech processing efficiency may lead to reduced understanding of words that then translates to reduced production of words, this project seeks to determine whether receptive vocabulary mediates (partially or completely explains) associations between P2 amplitude suppression and expressive vocabulary. In this statistical model, the direct effect of interest is the association between P2 amplitude suppression and expressive vocabulary. The indirect effect of interest includes (a) the relation between P2 amplitude suppression and receptive vocabulary and (b) the relation between receptive vocabulary and expressive vocabulary, controlling for P2 amplitude suppression (referred to as the “a path” and “b path,” respectively). If the product of these two relations is significant, we can conclude that P2 amplitude suppression “indirectly” impacts expressive vocabulary through receptive vocabulary, as hypothesized by the cascading effects framework. This study is the first, to our knowledge, to evaluate direct and indirect effects of P2 amplitude suppression on vocabulary as potential empirical support for the cascading effects framework in autistic children.

Our research team has developed a novel, low-demand ERP paradigm to measure amplitude suppression as a proxy of audiovisual speech processing efficiency in this population. In a pilot study investigating the psychometrics of indices derived from this ERP task, we found that the amplitude of one particular ERP component—the P2—in response to both auditory-only and audiovisual speech was stable in school-age children on the autism spectrum [53]. Notably, N1 amplitude and latencies for both N1 and P2 were not stable in school-age children [53], likely because the N1 component is not fully consolidated in early childhood and because ERP latencies are generally less stable than amplitudes [54,55]. The stability of any given variable places mathematical bounds on the variable’s validity to detect effects of interest [56,57]; thus, the present study focuses on the P2 amplitude in response to audiovisual versus auditory-only speech as a proxy of efficiency of audiovisual speech processing in autistic children and a control group of nonautistic peers.

### 1.2. Research Questions and Hypotheses

The purpose of this study was to (a) evaluate differences in audiovisual speech processing efficiency and (b) explore associations between the efficiency of audiovisual speech processing and vocabulary in autistic and nonautistic children. Our research questions were:

1. Is the efficiency of audiovisual speech processing—as approximated by P2 amplitude suppression—reduced, on average, in autistic children compared to their nonautistic peers?
2. Is the efficiency of audiovisual speech processing, as indexed by the degree of amplitude suppression displayed, positively associated with vocabulary in autistic and nonautistic children? Specifically, does the degree of amplitude suppression displayed indirectly influence expressive vocabulary through its relation with receptive vocabulary?

We hypothesized that autistic children would demonstrate reduced P2 amplitude suppression, on average, compared to their nonautistic peers and that P2 amplitude suppression would influence expressive vocabulary through receptive vocabulary, such that increased amplitude suppression would be associated with higher receptive vocabulary scores and, indirectly, with higher expressive vocabulary scores.

## 2. Materials and Methods

### 2.1. Design Overview

To answer these research questions, we conducted a study with intact-group and concurrent correlational design elements. All procedures were approved by the Institutional Review Board at Vanderbilt University Medical Center. The project was initially approved on 15 September 2010 (project no. 101194). Parents provided written informed consent, and participants provided written or verbal assent prior to participation in the study. All participants were financially compensated for their participation with gift cards.

### 2.2. Participants

Participants were 25 school-aged (i.e., 5.5–12.4 years old) autistic children and 25 nonautistic children matched at the group level on both chronological age and biological sex (see Table 1). Participants were drawn from a larger study of multisensory processing at Vanderbilt University; thus, our sample partially overlaps with previous reports from our team, i.e., [53,58,59]. A priori power analyses indicated that a sample of 50 total participants was sufficient to detect an indirect effect comprised of a and b paths that were moderate to large in magnitude (operationalized as *r* > 0.39 and 0.51 for moderate and large, respectively) [60].

The eligibility criteria for both groups were: (a) monolingual English speakers; (b) no history of seizure disorders; (c) normal or corrected-to-normal vision, as confirmed by screening via Pediatric Snellen and/or Tumbling “E” eye chart; (d) normal hearing, as confirmed by screening in a sound booth at 20 dB at octave intervals from 500 Hz to 4000 Hz, bilaterally; and (e) no diagnosed genetic disorders, such as fragile X, Down syndrome, or tuberous sclerosis. For the autism group, an additional inclusion criterion was a diagnosis of autism by a licensed clinician, confirmed by a research-reliable administration of the *Autism Diagnostic Observation Schedule*, second edition (ADOS-2; [64]), module 3 (*n* = 23) or module 2 (*n* = 2). Additional inclusion criteria for the nonautism group were (a) caregiver reports of autism symptoms below the screening threshold for autism concern on the Lifetime Version of the Social Communication Questionnaire [65]; (b) no immediate family history of autism; (c) no history or present indicators of learning or developmental disorders per parent report; and (d) nonverbal cognitive scores at or above 85 on the Leiter International Performance Scales, third edition (Leiter-3; [61]).

### 2.3. Stimuli

**Figure 2:**
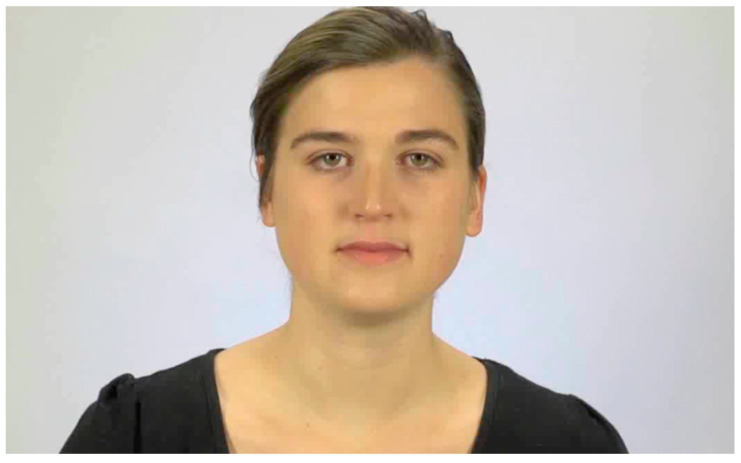
EEG stimuli consisted of the syllable “ba” spoken by a female speaker with neutral affect against a white background. The auditory-only condition consisted of the spoken syllable presented with a still image of the speaker’s face, while the audiovisual condition included the complementary mouth movements of the speaker.

Stimuli consisted of the consonant-vowel syllable “ba” as naturally spoken by an adult female speaker using a neutral facial expression against a white background (see Figure 2). In the audiovisual condition, the corresponding auditory and visual stimuli were presented in synchrony (i.e., with the visual cues from the face and neck temporally preceding the auditory cues in onset as naturally produced by the speaker). In the auditory-only condition, the auditory stimulus was presented with a still image of the speaker’s face to control for effects of the presence/absence of a face on speech processing. These stimuli have been utilized in several previous investigations in our laboratory [53,59]. During this video, participants were told that they were going to play a game wherein they needed to look for aliens that would only appear if they were still and quiet. Images of cartoon aliens were presented periodically between trials (i.e., after every fourth trial, there was a 50% chance of an alien image appearing) to maintain participant attention to the task. Participants were instructed to hit a BIGmack button (AbleNet Inc., Roseville, MN, USA) to “catch” the aliens each time one appeared on the screen.

### 2.4. Measurement of Efficiency of Audiovisual Speech Processing

EEG data were collected using Net Station EEG Software and a 128-channel Geodesic sensor net (Net Amps 400 amplifier, Hydrocel GSN 128 EEG cap, EGI Systems Inc., Eugene, OR, USA). The raw signal was sampled at 1000 Hz and referenced to vertex (Cz), and impedances were kept at or below 40 kΩs. Stimuli were presented via E-Prime in conjunction with an Eyelink1000 Plus eyetracker, which controlled stimulus presentation.

Prior to each run of the task, each participant’s eye gaze was calibrated using a five-point calibration procedure. Calibration was performed twice to validate the accuracy of the calibration. Before the first run of the task, participants viewed a video wherein a member of the research team described the task and a school-aged child modeled the task (i.e., wore the EEG cap and attended to the stimuli). During the task, 50 trials of each stimulus type (i.e., audiovisual and auditory-only, as described above) were presented in random order in two blocks for a total of 100 trials of each stimulus type across the two blocks. Trials were separated by an interstimulus interval (ISI) that was randomly jittered between 400 ms and 800 ms plus a 500 ms gaze contingency period, wherein the Eyelink1000 Plus sampled to ensure that the child was looking towards the monitor on which the visual stimulus was to be presented (i.e., minimum ISI between 900 ms and 1300 ms). Trials were initiated and data were presented via E-Prime once the specified gaze contingency was met. Between the two blocks, children took a scheduled break. The two blocks were completed in one recording session that was approximately 30 min in total duration.

### 2.5. Measurement of Receptive and Expressive Vocabulary

Concurrent receptive and expressive vocabulary was measured using the Receptive One-Word Picture Vocabulary Test, Fourth edition (ROWPVT [62]), and the Expressive One-Word Picture Vocabulary Test, Fourth edition (EOWPVT [63]), respectively. The ROWPVT and EOWPVT are norm-referenced across a wide-age range and appropriate for individuals with and without clinical diagnoses. The ROWPVT involves matching an English word to a picture of the object, action, or concept presented in multiple choice format, and the EOWPVT involves labeling objects, actions, and concepts presented in pictures [62,63]. Standard scores from these standardized and norm-referenced measures were derived according to the test manuals for use in analyses.

### 2.6. Data Processing and Analytic Plan

EEG data were bandpass-filtered from 0.5 Hz to 50 Hz, with a 6 dB roll-off of 0.25 Hz to 50.25Hz, using the EEGLAB firfiltnew.m function, and artifacts and bad channels were manually removed in EEGLAB [66]. An average of 138 trials (±34) were retained across stimulus types and participants; the autism and nonautism groups did not differ in the number of trials retained (*p* > 0.05). After the data were cleaned, they were re-referenced to the average, and removed channels were interpolated. Trials were baseline-corrected from 200 ms to 0 ms pre-stimulus onset. The mean amplitude of the P2 component (i.e., window defined a priori as occurring between 160 ms and 240 ms) as measured at Cz (i.e., electrode selected a priori, based on prior work using a similar task that identified P2 within the specified window at this location [67]) was extracted from the average waveform of each participant for each condition.

No data were missing in these analyses. To answer our first research question, P2 amplitudes in response to audiovisual versus auditory-only speech were compared using a 2 (Group) × 2 (Condition) ANOVA. Additionally, we calculated P2 amplitude suppression as the difference between the P2 amplitude in the auditory-only condition and in the audiovisual condition (auditory-only P2 amplitude–audiovisual P2 amplitude) for each participant; positive values indicated a greater degree of amplitude suppression (i.e., a more reduced audiovisual P2 amplitude compared to the auditory-only P2 amplitude). To answer our second research question, mediation analyses were carried out using PROCESS in R [68,69] to determine whether the association between P2 amplitude suppression as indexed by difference scores and expressive vocabulary standard scores was mediated by receptive vocabulary standard scores. As summarized above, in this mediation model the indirect effect comprised (a) the association between P2 amplitude suppression and receptive vocabulary (i.e., the a path) and (b) the association between receptive vocabulary and expressive vocabulary, controlling for P2 amplitude suppression (i.e., the b path). The product of the unstandardized coefficients for these two associations represents the indirect effect and is considered statistically significant when the bias-corrected bootstrap 95% confidence interval (CI) for the product of the unstandardized coefficients of the two paths does not include zero. The group was evaluated as a moderator. Outliers were monitored using both Cook’s D and centered leverage values; participants with values greater than three times the mean for both values across analyses were removed (*n* = 1 in the autism group).

## 3. Results

**Figure 3:**
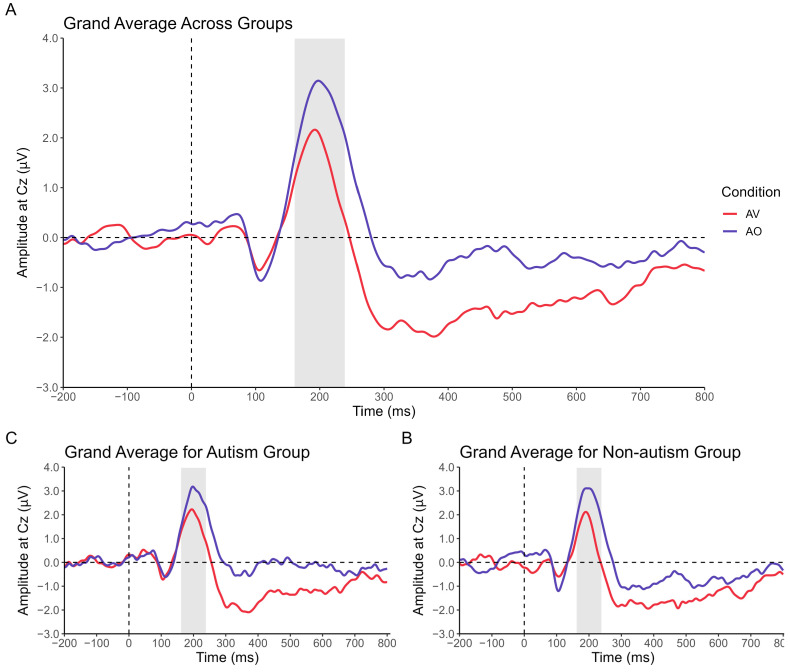
Grand average event-related potentials (ERPs) at Cz for (**A**) all participants, (**B**) the autism group, and (**C**) the nonautism group. Red = audiovisual (AV) speech condition; blue = auditory-only (AO) speech condition. Shaded region represents a-priori-defined window for P2 (160–240 ms).

See Table 1 for a summary of the participant characteristics. The groups significantly differed on nonverbal IQ and receptive vocabulary scores. The grand average ERPs, both across and within groups for each condition, are plotted in Figure 3.

### 3.1. Results Relevant to Research Question 1: Processing of Audiovisual and Auditory-Only Speech According to Group

**Figure 4:**
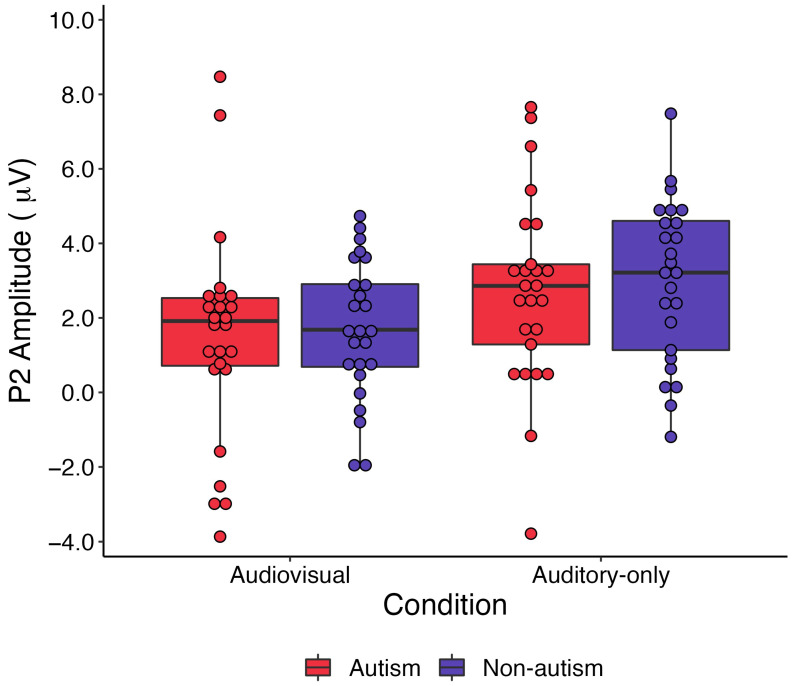
Results of the 2 (Group) × 2 (Condition) ANOVA. P2 amplitude was significantly reduced in response to audiovisual versus auditory-only speech across groups. There was neither a significant main effect of Group nor a significant Group × Condition interaction (*p* values > 0.6). Red = autism group; blue = nonautism comparison group.

Results of the 2 (Group) × 2 (Condition) ANOVA indicated a main effect of Condition, *F*(1,48) = 27.7, *p* < 0.001, $\eta_{p}^{2}$ = 0.366, whereby P2 amplitude was significantly reduced in response to audiovisual versus auditory-only speech, on average, across groups (see Figure 4). This statistically significant result was large in magnitude. There was not a significant between-group difference in P2 amplitude on average, *F*(1,48) = 1.93, *p* = 0.663, $\eta_{p}^{2}$ = 0.004, or a significant Group × Condition interaction, *F*(1,48) = 0.04, *p* = 0.867, $\eta_{p}^{2}$ = 0.001. Effects related to the group were negligible in magnitude.

### 3.2. Results Relevant to Research Question 2: Indirect Effect of P2 Amplitude Suppression on Vocabulary

**Figure 5:**
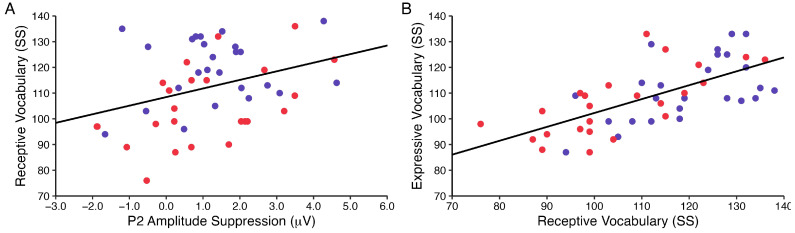
Results of the a and b paths of the mediation model. (**A**) P2 amplitude suppression difference scores were significantly positively associated with receptive vocabulary standard scores, and (**B**) receptive vocabulary standard scores were significantly positively associated with expressive vocabulary standard scores, controlling for P2 amplitude suppression. Red = autism group; blue = nonautism comparison group.

**Figure 6:**
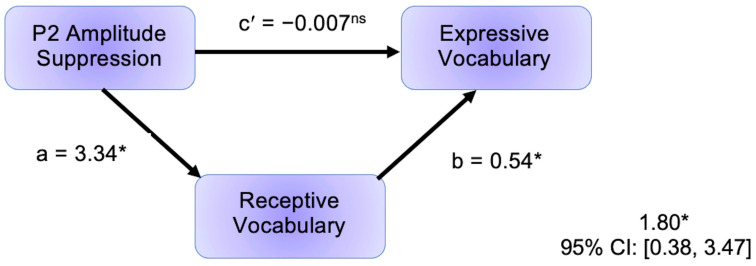
Full results of the mediation model, depicting the indirect effect of P2 amplitude suppression on expressive vocabulary through receptive vocabulary. Receptive vocabulary standard scores significantly mediated associations between P2 amplitude suppression and expressive vocabulary standard scores. * Path (or overall model, depicted bottom right) was statistically significant, *p* < 0.05 for a and b paths, 95% confidence interval does not include zero for overall model. ns = path was not statistically significant, *p* > 0.05.

P2 amplitude suppression was positively associated with receptive vocabulary (*r* = 0.344, *p* = 0.016), such that greater P2 amplitude difference scores were associated with higher receptive vocabulary standard scores. Additionally, receptive vocabulary was positively associated with expressive vocabulary, controlling for P2 amplitude suppression (*r* = 0.650, *p* < 0.001); see Figure 5. Thus, both the a and b paths comprising the indirect effect were statistically significant. The associations between P2 amplitude suppression and vocabulary scores did not vary according to group (*p* values for product terms in regression models testing moderated effects > 0.05). The mediation model indicated that receptive vocabulary scores significantly mediated associations between P2 amplitude suppression and expressive vocabulary scores (95% CI: [0.3838, 3.4715]). See Figure 6 for a depiction of the mediation model.

### 3.3. Post Hoc Analyses

Post hoc analyses assessed whether reductions in P2 amplitude in response to audiovisual versus auditory-only speech were moderated by chronological age and biological sex. These analyses showed that differences in P2 amplitude in response to audiovisual versus auditory-only speech did not vary according to chronological age or biological sex (*p* values for product terms in models testing moderated effects > 0.05).

Post hoc correlational analyses also evaluated whether P2 amplitudes in response to auditory-only and audiovisual speech alone (i.e., versus the differences scores intended to tap the increased efficiency of speech processing associated with having access to audiovisual versus auditory-only speech cues) were associated with receptive and expressive vocabulary standard scores. Auditory-only P2 amplitude was not significantly associated with receptive vocabulary (zero-order correlation = −0.019, *p* = 0.898) or expressive vocabulary (zero-order correlation = 0.012, *p* = 0.936). Audiovisual P2 amplitude was also not significantly associated with receptive vocabulary (zero-order correlation = −0.251, *p* = 0.082) or expressive vocabulary (zero-order correlation = −0.138, *p* = 0.345).

## 4. Discussion

This study employed a novel biobehavioral measure of speech processing to evaluate hypothesized links between an index of audiovisual speech processing efficiency and vocabulary in autistic and nonautistic children. The findings suggest that audiovisual speech processing is associated with vocabulary across groups. These results have implications for the cascading effects framework and underscore the role of speech processing in language ability in autistic and nonautistic children.

This study demonstrated that both autistic and nonautistic children, on average, display an increased efficiency of speech processing as approximated by P2 amplitude suppression in response to audiovisual versus auditory-only speech. Specifically, the mean P2 amplitude, which we have previously observed to be highly stable in school-age children on the autism spectrum [53], is smaller on average when children have access to visual speech cues that correspond to the auditory speech signal. These findings agree with prior reports of reduced P2 amplitude in response to audiovisual versus auditory-only speech in neurotypical children and adults [41,44,47] and provide increased empirical support for the use of amplitude suppression as a neural proxy of audiovisual speech processing in autistic and nonautistic children.

Interestingly, there was a lack of evidence for a between-group difference in the degree of P2 amplitude suppression experienced, on average. However, there were substantial individual differences in the degree of P2 amplitude suppression displayed across both autistic and nonautistic groups. Some children displayed a high degree of P2 amplitude suppression (i.e., a large reduction in P2 amplitude in response to audiovisual relative to auditory-only speech). Other children displayed little to no P2 amplitude suppression (i.e., similar P2 amplitudes in response to audiovisual relative to auditory-only speech). Still, others presented with an unexpected pattern marked by increased P2 amplitude for audiovisual relative to auditory-only speech. We hypothesize that such a pattern may reflect reduced processing efficiency in the presence of visual speech cues for some children, most likely the subset of children who display broader differences in the processing and perception of multisensory stimuli; additional work is needed to test this hypothesis. It is also possible that groups may differ on other EEG metrics that can be derived from speech or language tasks, including oscillatory activity or different ERP components. Future work should consider such indices in further characterizing speech processing in both autistic and nonautistic children.

Importantly, individual differences in P2 amplitude suppression covaried with vocabulary across groups. Specifically, P2 amplitude suppression was significantly and positively associated with receptive vocabulary, such that a greater degree of, or higher difference score indexing, amplitude suppression for audiovisual versus auditory-only speech tended to co-occur with higher receptive vocabulary. The relation between amplitude suppression and receptive vocabulary translated to expressive vocabulary, which is a more distal skill according to our theoretical framework (see Figure 1), as evidenced by a statistically significant indirect effect. These associations were not moderated by group. Notably, groups differed on receptive vocabulary scores, but not expressive vocabulary scores. It has been previously observed that autistic children can present with disproportionate deficits in receptive vocabulary, in some cases even having lower receptive vocabularies relative to their expressive vocabulary [70,71,72], although this unusual pattern is not universal. The current findings suggest that measures of efficiency of audiovisual speech processing may hold some promise for explaining phenotypic variance in receptive vocabulary, and indirectly in expressive vocabulary, in autistic and nonautistic children.

### Limitations and Future Directions

This study suggests that a novel biobehavioral index of audiovisual speech processing may explain variability in vocabulary in autistic and nonautistic children across a wide age range; however, it is not without limitations. First, this study may not generalize to all autistic and nonautistic children. Although our sample was heterogenous in regard to language and cognitive abilities, it did comprise a relatively small sample of individuals who were capable of following simple directions to wear an EEG cap, remain still, and actively attend to an audiovisual task. Additionally, the sample was more homogenous than we had hoped in regard to race and ethnicity, comprising primarily White and non-Hispanic children. Therefore, additional work is needed to evaluate the extent to which the present findings will generalize to the broader population of persons affected by autism. Conclusions are also limited by the lack of a visual-only condition in the task. A visual-only condition was omitted to maximize the number of trials presented to participants (and thereby the stability of scores) within conditions contributing the quantification of P2 amplitude suppression, as prior work indicated no P2-detectable response to visual-only speech at the Cz location of interest [38]. Although associations between audiovisual speech processing and vocabulary were established, it is not known whether autistic children would have differed in their responses to visual speech cues specifically or the degree to which visual speech processing alone is associated with vocabulary. The presentation of only one syllable, although selected carefully based on the presence of clear (bilabial) articulatory cues, may also limit the generalizability of results to other audiovisual speech stimuli. Furthermore, we only evaluated associations between efficiency of audiovisual speech processing and vocabulary via a concurrent correlational design. Therefore, we cannot draw conclusions regarding the directionality, or causality, of the observed relations. Future studies will provide much-needed insight into the degree to which the efficiency of audiovisual speech processing, as indexed by our innovative ERP task, may be useful for predicting the future vocabulary and broader spoken language ability of autistic and nonautistic children.

## 5. Conclusions

This study extends prior work on the indices of audiovisual speech processing efficiency in neurotypical persons by showing that P2 amplitude suppression is present, on average, across autistic and nonautistic children and associated with vocabulary across groups. Overall, the results suggest that P2 amplitude suppression may not differentiate autistic from nonautistic children, at least at school age, but may be a useful tool for explaining individual differences in language ability across autistic and nonautistic children. These findings provide increasing empirical support for the theory that disruptions in audiovisual processing and perception may underlie, or at least contribute to, language impairments in clinical populations [20], and warrant further research on audiovisual speech processing in childhood and its role as a neurobehavioral predictor of language and potential intervention target for autistic children.

## Figures and Tables

**Table 1:** Means and standard deviations of selected variables by group.

	Autism (n = 25) M (SD)	Nonautism (n = 25) M (SD)	*p* Value
Chronological Age (years)	8.9 (2.2) Range: 5.5–12.3	9.1 (2.2) Range: 5.8–12.4	0.698
Nonverbal IQ *	105.4 (10.8) Range: 75–126	116.2 (12.1) Range: 96–137	**0.002**
ADOS Calibrated Severity Score	8.2 (1.7) Range: 4–10	NA	NA
Receptive Vocabulary Age Equivalency (years) *	9.2 (3.3) Range: 4.9–18.6	11.9 (3.2) Range: 5.5–18.6	**0.006**
Expressive Vocabulary Age Equivalency (years)	9.7 (3.1) Range: 5.3–18.6	10.8 (3.5) Range: 5.0–17.9	0.219
Receptive Vocabulary Standard Score **	104.4 (14.92) Range: 76–136	119.4 (12.37) Range: 94–138	**<0.001**
Expressive Vocabulary Standard Score	106.2 (12.52) Range: 87–133	111.6 (12.33) Range: 87–133	0.131
Auditory-Only P2 Amplitude (μV)	2.7 (2.6) Range: −3.8–7.7	3.0 (2.2) Range: −1.2–7.5	0.638
Audiovisual P2 Amplitude (μV)	1.5 (2.9) Range: −3.9–8.5	1.7 (1.9) Range: −2.0–4.7	0.730

Note. *p* value = Significance level for *t*-test evaluating group difference. Nonverbal IQ was measured by the Leiter International Performance Scale, 3rd edition [61]. Receptive vocabulary was measured by the Receptive One-Word Picture Vocabulary Test [62]. Expressive vocabulary was measured by the Expressive One-Word Picture Vocabulary Test [63]. * Groups significantly differed, *p* < 0.05, *p* values bolded. ** Groups significantly differed, *p* < 0.001, *p* values bolded.

## Data Availability

The datasets analyzed for this study are available upon request.
